# Comparison of Th1/Th2 cytokine profiles between primary and secondary haemophagocytic lymphohistiocytosis

**DOI:** 10.1186/s13052-016-0262-7

**Published:** 2016-05-21

**Authors:** Yuanyuan Chen, Zhujun Wang, Zebin Luo, Ning Zhao, Shilong Yang, Yongmin Tang

**Affiliations:** Division of Hematology-oncology, Children’s Hospital of Zhejiang University School of Medicine, #57 Zhuganxiang Road, Hangzhou, 310003 PR China

**Keywords:** Haemophagocytic lymphohistiocytosis, Cytokines, Interleukin-4, Interferon-γ

## Abstract

**Background:**

Haemophagocytic lymphohistiocytosis (HLH) is a life-threatening disorder of immune regulation, and HLH patients with mutations in genes including *PRF1, UNC13D, STX11*, *STXBP2*, *SH2D1A, XIAP,* and *ITK* were reported to be primary HLH. Due to the different treatment options, the differentiation between primary and secondary HLH is critical. Our previous studies have showed that a Th1/Th2 cytokine profile is diagnostic for HLH, yet the cytokine profiles between primary and secondary HLH have not been compared. The aim of the study was to test whether the Th1/Th2 cytokine profile could be used as a tool to differentiate between primary and secondary HLH.

**Methods:**

A total of 45 hospitalized Chinese children with HLH during the period of February 2010 through September 2012 were enrolled in the study. Fifty healthy children were enrolled as controls. Primary HLH related genes were sequenced using genomic DNA samples. The Th1/Th2 cytokine levels including interferon-γ (IFN-γ), tumor necrosis factor-alpha (TNF-α), interleukin (IL)-10, IL-6, IL-4 and IL-2 were quantitatively determined by cytometric bead assay techniques.

**Results:**

Primary HLH group (*n* = 4) included one patient with biallelic heterozygous mutations in *PRF1* gene, and three patients with hemizygous mutation in *SH2D1A* gene. Based on the available genetic data, the other 41 patients were classified into the secondary HLH group. When compared the cytokine levels between the two groups, IL-4 level in primary-HLH was significantly lower than that in secondary HLH (*P* = 0.025), while IFN-γ level in primary HLH had a tendency of statistically lower than that in secondary HLH (*P* = 0.051). Area under receiver operating characteristic (ROC) curves of IL-4 and IFN-γ, IL-10, TNF-α, IL-2, and IL-6 levels were 0.841, 0.799, 0.506, 0.494, 0.457, and 0.250, respectively. ROC curves showed that 1.7 pg/ml of IL-4 had sensitivity and specificity for differentiation between primary and secondary HLH as 70.7 and 100.0 %, while 433.9 pg/ml of IFN-γ had sensitivity and specificity as 51.2 and 100.0 %, respectively.

**Conclusions:**

HLH patients with lower IL-4 and IFN-γ levels have higher possibility to be primary HLH. The cytokine profile may be used as an additional tool for the quick differential diagnosis between primary and secondary HLH.

**Electronic supplementary material:**

The online version of this article (doi:10.1186/s13052-016-0262-7) contains supplementary material, which is available to authorized users.

## Background

Haemophagocytic lymphohistiocytosis (HLH) is a life-threatening disorder of immune regulation, characterized by a highly stimulated, but ineffective immune response to antigens, which results in cytokine storm and inflammatory reaction [1]. HLH is not a single entity, but a clinical syndrome that can be encountered in association with various underlying diseases leading to similar characteristic clinical and laboratory presentations. Briefly, the diagnosis of HLH requires either a genetic diagnosis, or fulfillment of 5 out of 8 clinical criteria including fever, splenomegaly, bicytopenia, either hypofibrinoginemia or hypertriglyceridemia, hyperferritinemia, elevated soluble interleukin-2 receptor (sCD25 or sIL2R), impaired natural killer (NK) cell cytolytic function, or the observation of hemophagocytosis in bone marrow, spleen, or lymph nodes [2]. Based on the etiology, HLH can be classified into two types, primary HLH (with genetic mutations), and secondary HLH (no known mutations). Epstein–Barr virus (EBV) infections seem to be very common in HLH patients, especially in Asian countries [3–7]. Beside EBV infection, cytomegalovirus (CMV) infection is another common cause in HLH patients [8, 9]. Clinically, prompt and accurate diagnosis is critical to initiate definitive therapy. Unfortunately, the clinical features are identical in primary and secondary HLH, and both forms are often triggered by infections, so it is difficult to distinguish between these two types [8]. Furthermore, the diagnosis of HLH based on the current combination of clinical, laboratory and immunological criteria is challenging because all of the criteria are not specifically diagnostic for any HLH subtypes.

Primary HLH can be further divided into familial hemophagocytic lymphohistiocytosis (FHL) and immune deficiencies associated HLH [10]. Five genetic defects have been identified in FHL. A potential gene locus (FHL1) has been reported to be associated with HLH, but the specific gene involved has not yet been identified [11]. The first described FHL related gene is *PRF1* gene (FHL2) [12], a gene encodes perforin protein. The next identified cause of FHL (FHL3) is *UNC13D* gene [13], which encodes Munc13-4 protein. Later, mutations of *STX11* gene and *STXBP2* gene are found to be the causes of FHL4 [14] and FHL5 [15, 16], respectively. Immune deficiencies associated HLH occur with significant frequency in X-linked lymphoproliferative syndrome type 1 (XLP1) [17], XLP type 2 (XLP2) [18], and IL-2–inducible T cell kinase deficiency-associated lymphoproliferation [19], which are characterized by mutations in *SH2D1A*, *XIAP*, and *ITK* genes, respectively.

Hypercytokinemia is a hallmark of HLH. Henter JI et al. suggested that hypercytokinemia may be caused by a genetic defect in cytokine regulation as FHL patients showed high cytokine levels [20]. Using the rapid cytometric bead array (CBA) technique, a specific cytokine profile (significant increase of IFN-γ and IL-10, combined with a slightly increased level of IL-6) for childhood HLH was described by our group in 2008 [21]. In our clinical practice, this cytokine profile was helpful for the early diagnosis of HLH and for the differentiation from other disease entities [22, 23]. However, one limitation of our previous cytokine assay was that we did not perform genetic sequencing of HLH involved genes simultaneously. In this study, we tried to sequence *PRF1, UNC13D, STX11*, *STXBP2*, *SH2D1A, XIAP,* and *ITK* genes, together with cytokine determination for HLH patients to test whether cytokine profile could be used as a tool to distinguish between primary HLH and secondary HLH patients.

## Methods

### Patients

The diagnoses of all our patients were made based on the HLH-2004 criteria [2]. A cohort of 45 consecutively hospitalized HLH patients were enrolled in the study, which included 27 males and 18 females with a male-to-female ratio of 1.5:1, and a mean age of 3.7 years with a range of 8 days through 12.3 years. Patients who had been treated by steroids before referral to our hospital were excluded. A total of 50 unrelated healthy individuals (30 males, and 20 females) matched for age, and race were recruited as healthy controls. All the patients and healthy controls were from our hospital between February 2010 and September 2012. No HLH patient showed signs of mucocutaneous albinism. The study protocol was reviewed and approved by the Ethics Committee at Children’s Hospital Zhejiang University School of Medicine and written informed consents were obtained from all participants’ parents or guardians before this study.

### Genetic analysis

Genomic DNA was isolated from peripheral blood using QIAamp DNA Blood Mini Kit (Qiagen, Hilden, Germany). The coding regions and flanking intronic sequences of *PRF1, UNC13D, STX11, STXBP2, SH2D1A, XIAP,* and *ITK* genes, and the deep intronic sequences of intron 1 in *UNC13D* gene were amplified by a polymerase chain reaction (PCR) machine (BIO-RAD) in our laboratory, and the PCR products were sequenced by a DNA sequencer in Invitrogen Company (Shanghai, China). We analyzed *PRF1, UNC13D, STX11, STXBP2,* and *ITK* genes in all 45 patients and 50 controls, and all the male patients (*n* = 27) and male controls (*n* = 30) were sequenced in their *SH2D1A* and *XIAP* genes. The mutations were validated by re-sequencing an independent PCR-generated amplicon from the subjects. The variants were named according to the Human Genome Variation Society and journal requirements.

Database investigation and in silico prediction of mutations were checked, including the frequency of each variant in the general population investigated by Exome Aggregation Consortium (ExAC, Cambridge, MA, URL: http://exac.broadinstitute.org, March, 2016 accessed), previous reports of the same variants in the literature checked from Human Gene Mutation Database (HGMD, http://www.hgmd.cf.ac.uk) and Google Scholar (GS, https://scholar.google.com/), in silico prediction results by Polymorphism Phenotyping-2 (PolyPhen-2, http://genetics.bwh.harvard.edu/pph2/) and Sorting Intolerant From Tolerant (SIFT, http://sift.jcvi.org/). For PolyPhen-2, higher scores mean more deleterious. For SIFT, amino acids with probabilities < 0.05 were predicted to be deleterious.

### Cytokine determination

All 45 HLH patients and 50 controls were tested for the six cytokines including IL-2, IL-4, IL-6, IL-10, TNF-α, and IFN-γ. Peripheral blood samples were collected, transferred to a serum separating tube and centrifuged at 1000 g at 20 °C for 20 min after clotting. The serum was carefully harvested, and the determination of the cytokines was performed immediately, or if the situation was not so urgent, the aliquot was temporarily stored at 2 °C to 8 °C until analysis (usually within 12 h). Concentrations of the six cytokines aforementioned were quantitatively determined using the CBA Human Th1/Th2 Cytokine Kit II (BD Biosciences, San Jose, California) as described previously [21]. The minimum and maximum limits of detection for all six cytokines were 1 and 5000 pg/ml, respectively.

### Degranulation assays with flow cytometry

Degranulation assay were tested in 36 HLH patients and 43 controls. The assay was performed as previously described with modifications [24]. Peripheral blood mononuclear cells (PBMC) were isolated by Ficoll gradient centrifugation, rested in Iscove’s modified Dulbecco medium (IMDM, Invitrogen, Carlsbad, California, USA) supplemented with 10 % fetal bovine serum (FBS, Sijiqing, Hangzhou, China) at 37°C in a humidified atmosphere of 5 % CO_2_ for 2 h, and washed in 1 × PBS (phosphate buffer saline). K562 cells were obtained from the American Type Culture Collection (ATCC, Rockefeller, Maryland, USA) and cultured in RPMI1640 medium (Invitrogen, Carlsbad, California, USA) supplemented with 10 % FBS. All the antibodies including CD3-FITC, CD8-PerCP, CD56-APC, CD107a-PE (H4A3, IgG1), and isotype controls were purchased from Becton Dickinson (San Jose, CA, USA).

5 × 10^5^ PBMCs were mixed with 5 × 10^4^ sensitive target cells (K562) as stimulants in 12 flat bottom well plates and incubated for 2 h at 37°C. An equal volume of RPMI-1640 culture medium to replace the volume of K562 cells were used as negative control. Then the cells were stained with 10μl of CD3-FITC, 10μl of CD8-PerCP, 2.5μl of CD56-APC, and 10μl of CD107a-PE (isotype IgG1-PE as negative control) for 30 min at 4 °C in the dark. After two washes with 1 × PBS of the above samples, flow cytometric analyses were performed by a FACSCalibur cytometer (Becton-Dickinson, San Jose, CA, USA). CD3^-^CD56^+^ and CD3^+^CD8^+^ T cells were gated as NK cells and Cytotoxic T cells (CTLs), respectively. Data were acquired with CellQuest software (BD Bioscience).

### Clinical data

Information at diagnosis of age, sex, with fever or not, hemoglobin levels, platelets numbers, white blood cell counts, percentage of neutrophils, total neutrophil counts, percentage of lymphocytes, total lymphocyte counts, triglyceride levels, fibrinogen levels, LDH levels, ferritin levels, sIL2R levels, and sIL2R/ferritin ratio were collected. Quantitative real-time PCR was used to detect EBV-DNA and CMV-DNA copies in sera.

### Statistical analysis

Serum concentrations of individual cytokines, clinical data were compared between groups using the Mann-Whitney U test. A chi-square test was used to assess ratio differences between groups. Receiver operating characteristic (ROC) curves were derived from the cytokine levels for all HLH patients. In a ROC curve, the sensitivity and specificity of all six cytokines were calculated for the differentiation between primary and secondary HLH. All statistical analyses were performed using SPSS 17.0 software (SPSS Inc, Chicago, Illinois). A two-sided *P*-value <0.05 was considered to be statistically significant.

## Results

### Genetic results

The genetic findings of the 45 HLH patients were shown in Table 1, Additional file 1: Figure S1, Additional file 2: Figure S2, Additional file 3: Figure S3, Additional file 4: Figure S4, Additional file 5: Figure S5, and Additional file 6: Figure S6. All variants classified as pathogenic were not detected in the controls, while those classified as single nucleotide polymorphisms (SNPs) were found in both HLH patients and healthy controls. One patient with biallelic heterozygous mutations in *PRF1* gene (**P2**), and three patients with hemizygous mutation in *SH2D1A* gene (**P16**, **P17** and **P26**) were categorized into primary HLH group (*n* = 4). Based on the available genetic data, the other 41 patients were classified into the secondary HLH group, including nine HLH patients with single heterozygous mutation (mutations in patients **P6**, **P10**, and **P45** were already reported in literature, while mutations in patients **P1**, **P3**, **P4**, **P5**, **P7**, and **P11** were not reported before), three HLH patients with only SNPs (**P8**, **P9**, and **P38**), and the remaining 29 HLH patients without any SNPs.

**Table 1:** Summary of genetic findings in 45 HLH patients

Case	Gender	Age	Candidate gene	Exon/intron	Nucleotide change	Amino acid change	Genotype
P2	F	6Y3M	*PRF1*	Exon3	c.757G>A c.1061A>T	p.Glu253Lys p.Asp354Val	heterozygous heterozygous
P16	M	3Y3M	*SH2D1A*	Exon2	c.191G>A	p.Trp64Ter	hemizygous
P17	M	11M24D	*SH2D1A*	Exon2	c.162C>G	p.Tyr54Ter	hemizygous
P26	M	11M17D	*SH2D1A*	Exon2	c.163C>T	p.Arg55 Ter	hemizygous
P1	M	1Y5M	*PRF1*	Exon2	c.385T>A	p.Trp129Arg	heterozygous
P3	M	4Y5M	*UNC13D*	Exon6	c.478G>A	p.Val160 Met	heterozygous
P4	M	2Y8M	*UNC13D*	Exon6	c.518C>T	p.Thr173Met	heterozygous
P5	F	1Y7M	*UNC13D*	Exon10	c.760C>T	p.Arg254Cys	heterozygous
P6	M	8D	*UNC13D*	Exon23	c.2296C>T	p.Glu766Ter	heterozygous
P7	M	25D	*UNC13D*	Exon32	c.3259C>T	p.Arg1087Trp	heterozygous
			*XIAP*	Exon5	c.1268A>C	p.Gln423Pro^a^	hemizygous
P8	M	7Y9M	*STXBP2*	Exon7	c.497C>T	p.Thr166Met^a^	heterozygous
P9	M	11Y11M	*STXBP2*	Exon7	c.497C>T	p.Thr166 Met^a^	heterozygous
P10	F	5Y8M	*STXBP2*	Exon7	c.575G>A	p.Arg192His	heterozygous
P11	M	1Y1M	*STXBP2*	Exon9	c.767T>C	p.Leu256Pro	heterozygous
P38	M	1Y1M	*XIAP*	Exon5	c.1268A>C	p.Gln423Pro^a^	hemizygous
P45	M	4Y	*UNC13D*	Intron1	c.118-307G>A	Unknown	heterozygous

Gender, *M* male, *F* female

Age, *Y* year, *M* month, *D* day

^a^Single nucleotide polymorphism (SNP)

*Ter* Terminator, which would result in truncated protein

Mutations in *PRF1, UNC13D, STX11, STXBP2, SH2D1A, XIAP,* and *ITK* genes of all the 45 HLH cases accounted for 2/45 (4.4 %), 6/45 (13.3 %), 0/45 (0.0 %), 2/45 (4.4 %), 3/45 (6.7 %), 0/45 (0 %), and 0/45 (0.0 %), respectively. For the four primary HLH cases, mutations in *PRF1* and *SH2D1A* genes accounted for 1/4 (25 %) and 3/4 (75 %), respectively. Three missense heterozygous variants in *PRF1* gene were found in one male and one female (Additional file 1: Figure S1). One heterozygous c.385T>A (p.Trp129Arg) mutation was found in **P1**, a 1-year-5-month male, while compound heterozygous c.757G>A (p.Glu253Lys) and c.1061A>T (p.Asp354Val) in *PRF1* gene were found in **P2**, a 6-year-3-month female, with c.757G>A inherited from her father and c.1061A>T from her mother. Five missense mutations of *UNC13D* gene (Additional file 2: Figure S2), c.478G>A (p.Val160 Met) in **P3**, c.518C>T (p.Thr173Met) in **P4**, c.760C>T (p.Arg254Cys) in **P5**, c.3259C>T (p.Arg1087Trp) in **P7**, and c.118-307G>A in **P45**, were found in four males and one female, respectively. Another heterozygous mutation c.2296C>T (p.Glu766Ter) in exon23 of UNC13D gene of an 8-day male patient (**P6**), with the cDNA study revealed that this mutation had a deleterious effect on splicing, c.2295_2298delGCAG (p.Glu765Aspfs*27) (Additional file 6: Figure S6), was already reported by our group as a special case [25]. Two heterozygous mutations of STXBP2 gene, c.575G>A (p.Arg192His), and c.767T>C (p.Leu256Pro), were identified in two patients (Additional file 3: Figure S3). Three hemizygous mutations of *SH2D1A* gene, c.162C>G (p.Tyr54Ter), c.163C>T (p.Arg55Ter), and c.191G>A (p.Trp64Ter), were identified in three male patients (Additional file 4: Figure S4). Two male patients (**P8** and **P9**) with c.497C>T (p.Thr166Met, SNP rs181216956) of *STXBP2* gene, and two male patients (**P7** and **P38)** with c.1268A>C (p.Gln423Pro, SNP rs5956583) of *XIAP* gene, were identified in this cohort (Additional file 5: Figure S5). No mutation was detected from *STX11* and *ITK* genes in all 45 HLH patients.

Database investigation and in silico prediction of mutations in HLH patients were shown in Table 2. The in silico prediction results between PolyPhen-2 and Sorting Intolerant From Tolerant (SIFT) were consistent with each other, except p.Arg1087Trp of *UNC13D* gene (PolyPhen-2 predicted this change was BENIGN with a score of 0.002, while SIFT indicated that this change would AFFECT PROTEIN FUNCTION with a score of 0.00).

**Table 2:** Database investigation and in silico prediction of mutations in 45 HLH patients

Case	Candidate gene	Nucleotide change	Amino acid change	ExAC allele frequency	HGMD/GS references	Polyphen-2	SIFT	CD107a
P2	*PRF1*	c.757G>A c.1061A>T	p.Glu253Lys p.Asp354Val	0.00003296 Not reported	[33] No reference	PROBABLY DAMAGING with a score of 0.962 POSSIBLY DAMAGING with a score of 0.952	AFFECT PROTEIN FUNCTION with a score of 0.01 AFFECT PROTEIN FUNCTION with a score of 0.02	32.28
P16	*SH2D1A*	c.191G>A	p.Trp64Ter	Not reported	[34, 35]	Not Available	Not Available	33.83
P17	*SH2D1A*	c.162C>G	p.Tyr54 Ter	Not reported	[36]	Not Available	Not Available	0.72
P26	*SH2D1A*	c.163C>T	p.Arg55 Ter	Not reported	[36, 37]	Not Available	Not Available	No done
P1	*PRF1*	c.385T>A	p.Trp129Arg	Not reported	No reference	PROBABLY DAMAGING with a score of 1.000	AFFECT PROTEIN FUNCTION with a score of 0.00	3.24
P3	*UNC13D*	c.478G>A	p.Val160 Met	Not reported	No reference	PROBABLY DAMAGING with a score of 0.999	AFFECT PROTEIN FUNCTION with a score of 0.05	35.14
P4	*UNC13D*	c.518C>T	p.Thr173Met	0.00001670	No reference	PROBABLY DAMAGING with a score of 1.000	AFFECT PROTEIN FUNCTION with a score of 0.01	0.43
P5	*UNC13D*	c.760C>T	p.Arg254Cys	0.0003450	No reference	PROBABLY DAMAGING with a score of 0.987	AFFECT PROTEIN FUNCTION with a score of 0.02	5.69
P6	*UNC13D*	c.2296C>T	p.Glu766 Ter	Not reported	[25, 38, 39]	Not Available	Not Available	0.51
P7	*UNC13D*	c.3259C>T	p.Arg1087Trp	0.0001438	No reference	BENIGN with a score of 0.002	AFFECT PROTEIN FUNCTION with a score of 0.00	2.64
	*XIAP*	c.1268A>C	p.Gln423Pro	0.3334	[40–42]	BENIGN with a score of 0.002	TOLERATED with a score of 0.30	
P8	*STXBP2*	c.497C>T	p.Thr166Met	0.0002454	No reference	BENIGN with a score of 0.022	TOLERATED with a score of 0.10	3.51
P9	*STXBP2*	c.497C>T	p.Thr166 Met	0.0002454	No reference	BENIGN with a score of 0.022	TOLERATED with a score of 0.10	13.37
P10	*STXBP2*	c.575G>A	p.Arg192His	Not reported	[15]	PROBABLY DAMAGING with a score of 1.000	AFFECT PROTEIN FUNCTION with a score of 0.00	13.73
P11	*STXBP2*	c.767T>C	p.Leu256Pro	Not reported	No reference	PROBABLY DAMAGING with a score of 1.000	AFFECT PROTEIN FUNCTION with a score of 0.00	4.7
P38	*XIAP*	c.1268A>C	p.Gln423Pro	0.3334	[40–42]	BENIGN with a score of 0.002	TOLERATED with a score of 0.30	0.07
P45	*UNC13D*	c.118-307G>A	Unknown	Not reported	[29, 39, 43]	Not Available	Not Available	3.07

*ExAC* Exome Aggregation Consortitium, *HGMD* Human Gene Mutation Database, *GS* Google Scholar

*PolyPhen-2* Polymorphism Phenotyping-2, *SIFT* Sorting Intolerant From Tolerant

*Ter* Termination, which would result to truncated protein

### Cytokine levels

**Fig. 1:**
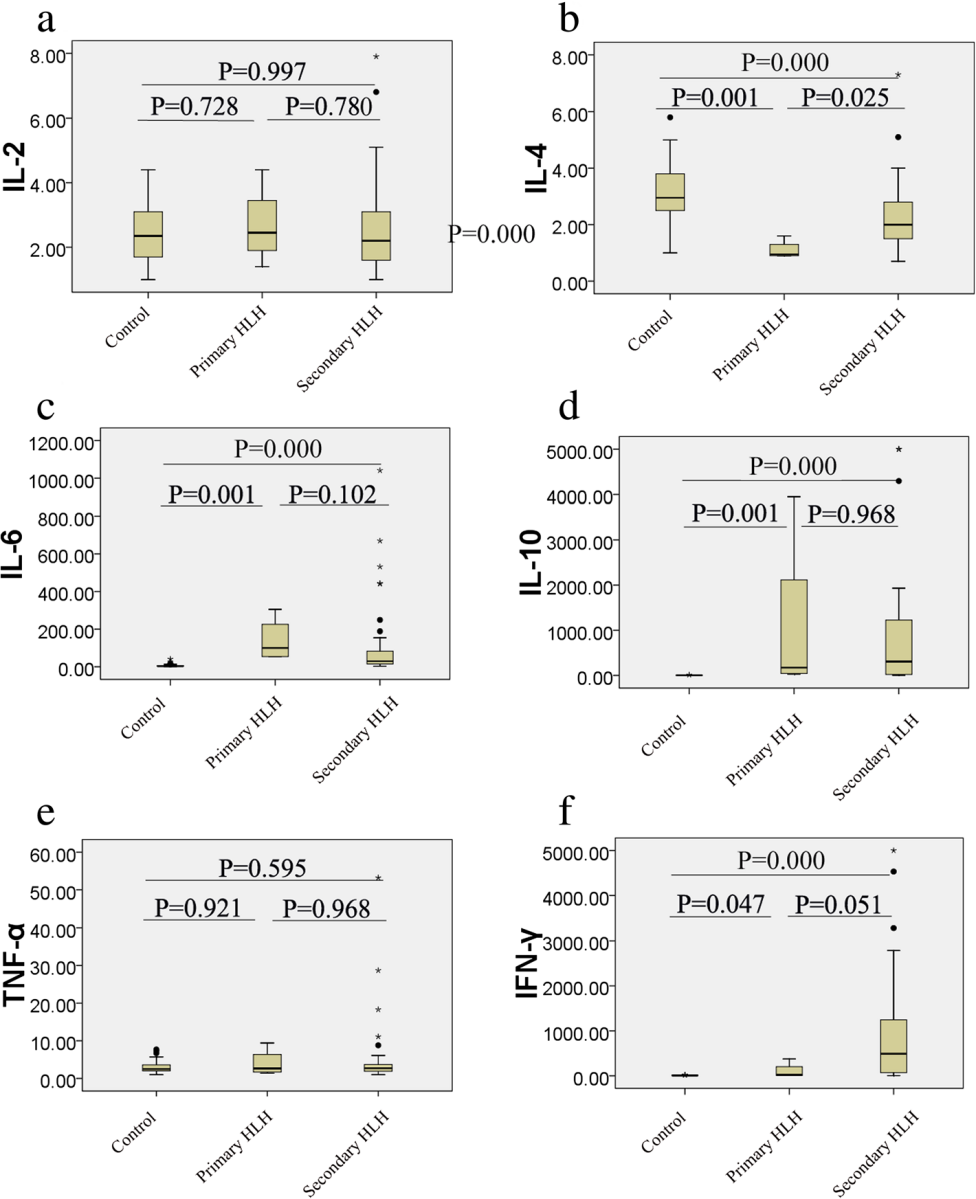
Comparisons of serum cytokine concentrations (pg/ml) among control, primary HLH, and secondary HLH. **a** IL-2; **b** IL-4; **c** IL-6; **d** IL-10; **e** TNF-α; **f** IFN-γ. The center horizontal line of the central box is the median (50th percentile), the bottom and top of the box are the 25th and 75th percentiles. The whiskers extend from each end of the box to the 5th and 95th percentiles of the values, respectively. Outliers are the data with values beyond the 5th and 95th percentiles

The mean (95 % Confidence Interval, CI) of serum IL-2, IL-4, IL-6, IL-10, TNF-α, and IFN-γ concentrations for healthy controls were 2.4 (2.2–2.7), 3.2 (2.9–3.5), 6.0 (4.0–7.9), 3.4 (3.0–3.9), 2.9 (2.5–3.4), 9.2 (8.3–10.0) pg/ml, respectively. As compared to control group, the levels of IL-4 in both primary and secondary HLH groups were significantly lower (*P* < 0.05) while those of IL-6, IL-10 and IFN-γ were significantly higher (*P* < 0.05) (Fig. 1). However, the levels of IL-2 and TNF-α were not statistically different among the three groups (all *P* > 0.05).

When we compared the levels of the cytokines between the primary and secondary HLH groups, the IL-4 level in primary-HLH was significantly lower than that in secondary HLH (*P* = 0.025), with mean (95 % CI) in primary and secondary HLH groups showed as 1.1 (0.6–1.6) and 2.3 (1.9–2.7), respectively. Additionally, IFN-γ level in primary HLH had a tendency of statistically lower than that in secondary HLH (*P* = 0.051), with mean (95 % CI) in primary and secondary HLH groups showed as 106.6 (minus-393.9) pg/ml and 905.7 (530.7–1280.6) pg/ml, respectively. The 95 % CI gap of IL-4 between primary and secondary HLH groups was 1.6–1.9 pg/ml, while the gap of IFN-γ between the two groups was 393.9–530.7 pg/ml. The levels of the remaining four cytokines including IL-2, IL-6, IL-10, and TNF-α were not significantly different between primary and secondary HLH groups (*P* = 0.78, *P* = 0.102, *P* = 0.968, and *P* = 0.968, respectively).

**Fig. 2:**
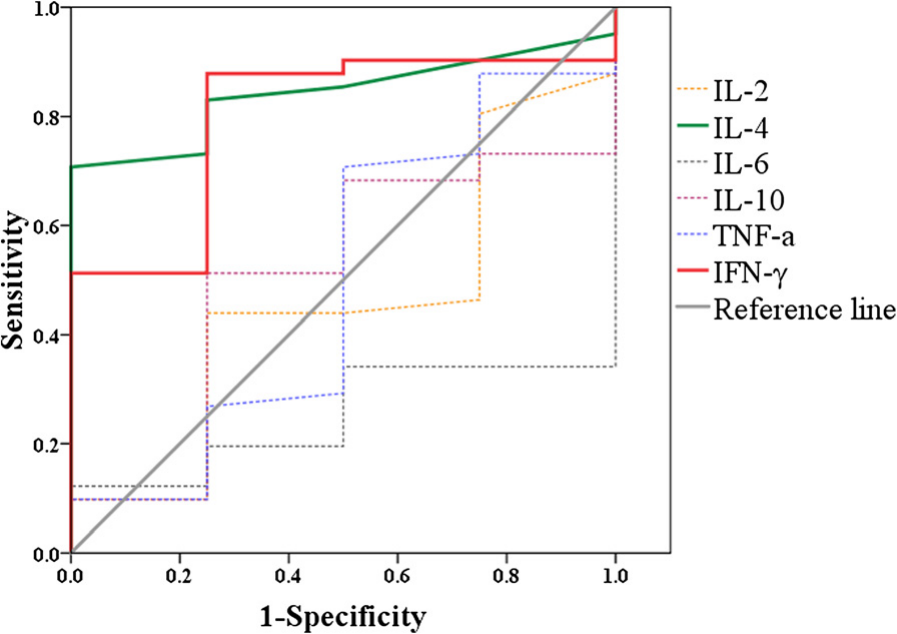
ROC curves of IL-2, IL-4, IL-6, IL-10, TNF-α, and IFN-γ between primary HLH and secondary HLH. The diagonal line is the reference line

The area under the ROC curve, referred to as the AUC, is an appropriate measure for describing the overall accuracy of a diagnostic test, and higher AUC value mean better diagnostic value. AUCs of IL-4, IFN-γ, IL-10, TNF-α, IL-2, and IL-6 levels were calculated to be 0.841, 0.799, 0.506, 0.494, 0.457, and 0.250, respectively, indicating that levels of IL-4 and IFN-γ may be used as additional tools for the quick differential diagnosis between primary and secondary HLH (Fig. 2). ROC curves of IL-4 levels showed that 1.6 pg/ml, 1.7 pg/ml, 1.8 pg/ml, and 1.9 pg/ml had sensitivity and specificity as 73.2 and 75.0 %, 70.7 and 100.0 %, 68.3 and 100.0 %, 63.4 and 100 %, respectively. Furthermore, ROC curves of IFN-γ levels showed that 360.6 pg/ml, 433.9 pg/ml, and 539.7 pg/ml had sensitivity and specificity as 51.2 and 75.0 %, 51.2 and 100.0 %, 48.8 and 100 %, respectively. Taken the results of IL-4 and IFN-γ together, we proposed that HLH patients with IL-4 below 1.7 pg/ml and IFN-γ below 433.9 pg/ml had a higher chance to be primary HLH.

### Degranulation and clinical data

Information of surface CD107a level in resting NK cells, age at diagnosis, sex, with fever or not, hemoglobin levels, platelet counts, white blood cell counts, percentage of neutrophils, absolute neutrophil counts, percentage of lymphocytes, absolute lymphocyte counts, triglyceride levels, fibrinogen levels, LDH levels, ferritin levels, sIL2R levels, sIL2R/ferritin ratio, EBV-DNA, and CMV-DNA copies were shown in Table 3. The results showed that, except the percentage of neutrophils (*P* = 0.012) and the percentage of lymphocytes (*P* = 0.012), there was no significant difference between primary HLH and secondary HLH groups in any other factors.

**Table 3:** Clinical information on 45 HLH patients

	Total (*n* = 45)	Primary (*n* = 4)	Secondary (*n* = 41)	*P*-value
Age at diagnosis				
≤12 months	9/45	2/4	7/41	0.116
>12 months	36/45	2/4	34/41	
Mean age (year, range)	3.7 (0–12.3)	2.9 (1.0–6.3)	3.8 (0–12.3)	0.646
Sex (M/F)	27/18	3/1	24/17	0.521
Fever	45/45	4/4	41/41	
Hemoglobin (<90 g/L)	16/45	1/4	15/41	0.644
Mean (g/L, range)	94.0 (46.0–135.0)	93.8 (84.0–102.0)	94.0 (46.0–135.0)	0.905
Platelets (<100x10^9^/L)	30/45	2/4	28/41	0.459
Mean (100x10^9^/L, range)	82.5 (32.0–400.0)	100.0 (5.0–215.0)	80.8 (3.0–400.0)	0.661
White blood cells				
Mean (1x10^9^/L, range)	7.5 (0.3–41.3)	6.5 (1.0–9.7)	7.6 (0.3–41.3)	0.842
Percentage of neutrophils				
Mean (%, range)	38.2 (2.0–81.5)	12.8 (6.9–19.4)	40.7 (2.0–81.5)	0.012
Total neutrophils (<1x10^9^/L)	20/45	2/4	18/41	0.815
Mean (1x10^9^/L, range)	2.9 (0.1–22.1)	0.9 (0.1–1.3)	3.1 (0.1–22.1)	0.413
Percentage of lymphocytes				
Mean (%, range)	50.4 (7.9–88)	75.3 (71.8–77.7)	48.0 (7.9–88.0)	0.012
Total lymphocytes				
Mean (1x10^9^/L, range)	3.6 (0.2–28.1)	4.9 (0.7–7.5)	3.4 (0.2–28.1)	0.175
Triglycerides (>3.0 mmol/L)	15/45	3/4	12/41	0.064
Mean (mmol/L, range)	2.7 (0.8–8.9)	3.0 (1.3–4.1)	2.7 (0.8–8.9)	0.425
Fibrinogen (<1.5 g/L)	30/45	4/4	26/41	0.138
Mean (g/L, range)	1.5 (0.2–4.9)	0.9 (0.5–1.2)	1.5 (0.2–4.9)	0.14
LDH (>500 IU/L)	32/45	3/4	29/41	0.857
Mean (IU/L, range)	1018.0 (167.0–6565.0)	814.0 (363.0–1644.0)	1037.9 (167.0–6565.0)	0.842
Ferritin (>500 μg/L)	45/45	4/4	41/41	
Mean (μg/L, range)	1477.7 (895.0–1500.0)	1500.0 (1500.0–1500.0)	1474.2 (895.0–1500.0)	0.655
sIL2R (≥2400 U/ml)	26/27	4/4	22/23	0.671
Mean (U/ml, range)	34092.7 (155.6–85787.7)	27198.1 (14250.9–38203.1)	35239.6 (155.6–85787.7)	0.922
sIL2R/Ferritin				
Mean (U/pg, range)	23.0 (0.1–57.2)	18.4 (9.5–25.5)	23.8 (0.1–57.2)	0.811
Degranulation (CD107a <5 %)	16/36	1/3	15/33	0.795
Mean (%, range)	10.3 (0.1–35.1)	22.3 (0.7–33.8)	9.2 (0.1–35.1)	0.407
EBV-DNA (>1000 copies)	26/45	2/4	24/41	0.741
CMV-DNA (>1000 copies)	1/45	0/4	1/41	0.752

*P* value: Comparisons between primary and secondary HLH groups

**Fig. 3:**
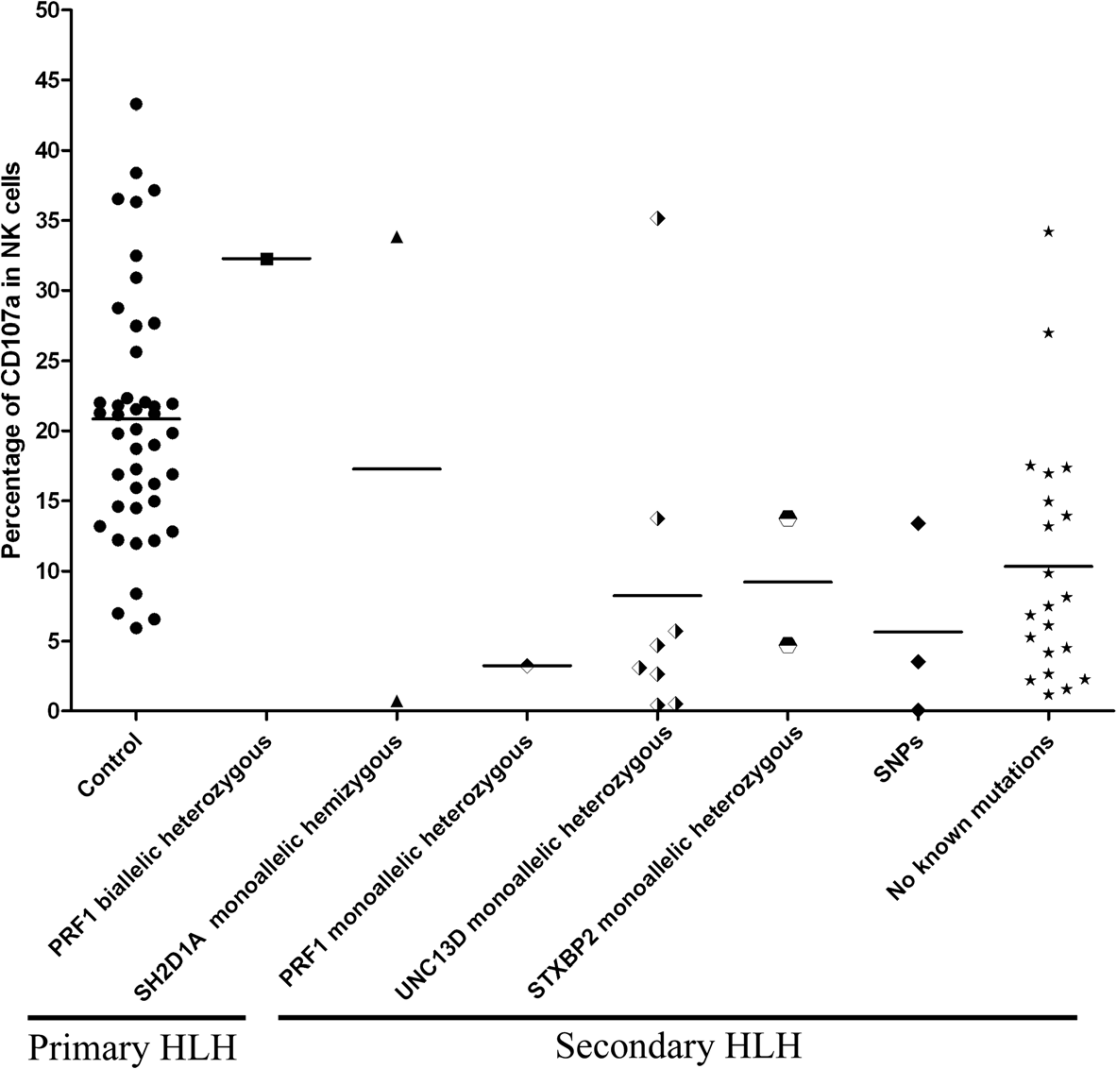
Degranulation results of healthy controls, primary HLH cases, and pecondary HLH cases. Shapes represent individual subjects, while horizontal bars show the mean in each group

The degranulation assay results in healthy controls, primary HLH cases, and secondary HLH cases were shown in Fig. 3, with mean (95 % CI) in controls was 20.9 (18.19–23.6) %. We defined resting NK cell degranulation below 5 % as defective [26]. The results showed 0/1 (0 %) patient with FHL2, 1/3 (33.3 %) patient with XLP, and 15/33 (45.5 %) patients with a diagnosis of secondary HLH had resting NK degranulation below 5 % (Additional file 7: Table S1). There was a high possibility that patients with defective degranulation could carry undetected mutations.

Meanwhile, we re-grouped HLH patients based on their CD107a levels in resting NK cells: HLH with CD107a<5 % (*n* = 16), and HLH with CD107a>5 % (*n* = 20). Comparisons of serum cytokine concentrations (pg/ml) among Control, HLH with CD107a<5 %, and HLH with CD107a>5 % were shown in Additional file 8: Figure S7, indicated similar results to primary and secondary HLH grouping. Comparing to the healthy control group, both HLH with CD107a<5 % and CD107a>5 % had higher IL-6, IL-10 and IFN-γ levels, and lower IL-4 level (with all *P* < 0.05). Between HLH with CD107a<5 % and CD107a>5 % groups, all six cytokines showed no statistical significance.

## Discussion

Inheritance of HLH is known to be autosomal recessive. Based on the data from published studies, primary HLH usually includes patients with homozygous, hemizygous, or compound heterozygous mutations, as mutations of these types affect protein function in a known way. In our HLH patients, no homozygous mutation was detected, and all were hemizygous or heterozygous variants. Similar to our results, Zhizhuo H et al. investigated 67 Chinese HLH patients and identified eight patients with variants in primary HLH related genes, included one patient with a hemizygous mutation of *XIAP* gene, three patients with compound heterozygous mutations, and four patients with single heterozygous mutation [27]. How does the single heterozygous mutation affect its coding protein’s function is complicated and remains to be elucidated. Based on the recent study of Spessott WA et al. in 2015 [28], some single heterozygous mutation in special site can cause primary HLH, yet the dominant negative effect can not be generalized, and people should do further study to prove the related protein function is damaged if they want to assign some HLH patients with monoallelic mutation as primary HLH. Another possibility is that the risk of having missed pathogenic variants is high, and possible additional variants, such as deep intronic variants similar to mutations in deep intron 1 of *UNC13D* gene [29–31], might have been missed for some cases.

In our previous study, we found that HLH patients presented a specific cytokine profile of highly increased levels of IFN-γ and IL-10, and a moderately increased level of IL-6 [23]. In this study, the levels of IL-6, IL-10, and IFN-γ in both primary and secondary HLH groups were significantly higher (*P* < 0.05) than those of controls, and no patient was overlapped with our previous study, which further confirmed the usefulness of this cytokine profile for the diagnosis of HLH.

When we compared the levels of the cytokines between the primary and secondary HLH groups, the IL-4 level in primary-HLH was significantly lower than that in secondary HLH (*P* = 0.025), with the gap of IL-4 between the two groups was 1.6–1.9 pg/ml, and IFN-γ level in primary HLH had a tendency of statistically lower than that in secondary HLH (*P* = 0.051), with the gap of IFN-γ between the two groups was 393.9–530.7 pg/ml. AUCs of six cytokines indicated that levels of IL-4 and IFN-γ may be used as additional tools for the quick differential diagnosis between primary and secondary HLH. Taken the results of IL-4 and IFN-γ together, we propose that HLH patients with IL-4 below 1.7 pg/ml and IFN-γ below 433.9 pg/ml have a higher possibility to be primary HLH.

There have been studies trying to find other discriminators to differentiate primary from secondary cases of HLH. According to the report by Bryceson et al. [26], degranulation assay has a high sensitivity and specificity rate for discrimination between two types of HLH (type1 includes FHL3, FHL4, and FHL5, while type2 includes FHL2, XLP1, XLP2, and secondary HLH cases). In this cohort, all primary HLH cases belong to type2 HLH including FHL2 and XLP1, and it is not surprising that we can not find difference of degranulation between primary HLH and secondary HLH groups. Yasumi et al. proposed that the percentage of total lymphocytes, serum levels of LDH, and the sIL2R/ferritin ratio could differentiate familial from secondary HLH [32]. In this study, when we compare to the secondary HLH patients, primary HLH patients have higher percentage of lymphocytes (mean level 75.3 % vs 48.0 %, *P* = 0.012), and lower percentage of neutrophils (12.8 % vs 40.7 %, *P* = 0.012), which is consistent with Yasumi et al’s findings.

Our study has some limitations. First, the risk of having missed pathogenic variants is high, and possible additional variants, such as deep intronic variants, regulatory variants, and complex structural variants, might have been missed for some cases, which would have great impact on the grouping strategy and then influence the interpretation of the results. Second, as this is a single-center study, and the results are based on data from small number of samples and incomplete genetic sequencing results, a multi-center study was required to be performed to validate the results.

## Conclusions

Among 45 HLH patients, four HLH patients are identified as primary HLH with hemizygous or compound heterozygous mutations, and 41 HLH patients belong to secondary HLH. Variants in *PRF1, UNC13D, STX11, STXBP2, SH2D1A, XIAP,* and *ITK* genes account for 2/45 (4.4 %), 6/45 (13.3 %), 0/45 (0.0 %), 2/45 (4.4 %), 3/45 (6.7 %), 0/45 (0 %), and 0/45 (0.0 %), respectively. HLH patients with lower IL-4 level, lower IFN-γ level, higher percentage of lymphocytes, and lower percentage of neutrophils have higher possibility to be primary HLH. The cytokine profiles can be used as an additional tool for the quick differential diagnosis between primary and secondary HLH.

## Additional files

Additional file 1: Figure S1. Sequencing results of *PRF1* gene. Genomic DNA sequencing results showed a 1-year-5-month male (**P1**) had heterozygous mutation c.385T>A (p.Trp129Arg), and a 6-year-3-month female (**P2**) had compound heterozygous c.757G>A (p.Glu253Lys) and c.1061A>T (p.Asp354Val) of *PRF1* gene. (TIF 1391 kb) Additional file 2: Figure S2. Sequencing results of *UNC13D* gene. Five missense mutations of *UNC13D* gene, c.478G>A (p.Val160 Met) in **P3**, c.518C>T (p.Thr173Met) in **P4**, c.760C>T (p.Arg254Cys) in **P5**, c.3259C>T(p.Arg1087Trp) in **P7**, and c.118-307G>A in **P45**, were found in four males and one female, respectively. (TIF 171 kb) Additional file 3: Figure S3. Sequencing results of *STXBP2* gene. Two mutations of *STXBP2* gene, c.575G>A (p.Arg192His) in **P10**, and c.767T>C (p.Leu256Pro) in **P11**, were found in one male and one female, respectively. (TIF 985 kb) Additional file 4: Figure S4. Sequencing results of *SH2D1A* gene. Three hemizygous mutations of *SH2D1A* gene, c.191G> (p.Trp64Ter) in **P16**, c.162C>G(p.Tyr54Ter) in **P17**, and c.163C>T(p.Arg55Ter) in **P26**, were identified in three male patients, respectively. (TIF 136 kb) Additional file 5: Figure S5. SNPs of *STXBP2* and *XIAP* genes. Two males patients, 7-year-9-month (**P8**) and 11-year-11-month (**P9**), showed c.497C>T (p.Thr166Met) in *STXBP2* gene, numbered SNP rs181216956; of *XIAP* gene, two male patients (**P7** and **P38**) were identified with c.1268A>C(p.Gln423Pro), numbered SNP rs5956583. (TIF 938 kb) Additional file 6: Figure S6. Sequencing results of *UNC13D* gene in a Chinese male neonate(**P6**) and his parents. In mRNA level of **P6**, the result manifested a heterozygous frameshift mutation c.2295_2298delGCAG (**A**), which was consistent with the heterozygous point mutation c.2296C>T in genomic DNA level (**B**). Sequencing results from cDNA and genomic DNA of **P6** and his parents both showed that **P6** inherited this mutation from his mother. (TIF 391 kb) Additional file 7: Table S1. Percentage of CD107a expression in NK cells from 43 controls and 36 HLH patients. (DOCX 15 kb) Additional file 8: Figure S7. Comparisons of serum cytokine concentrations (pg/ml) among control, HLH with CD107a<5 %, and HLH with CD107a>5 %. **A**: IL-2; **B**: IL-4; **C**: IL-6; **D**: IL-10; **E**: TNF-α; **F**: IFN-γ. The center horizontal line of the central box is the median (50th percentile), the bottom and top of the box are the 25th and 75th percentiles. The whiskers extend from each end of the box to the 5th and 95th percentiles of the values, respectively. Outliers are the data with values beyond the 5th and 95th percentiles. (TIF 957 kb)

## Abbreviations

95 % CI: 95 % Confidence Interval
AUC: area under ROC curve
CBA: cytometric bead assay
CTLs: cytotoxic T cells
ExAC: Exome Aggregation Consortitium
FHL: familial hemophagocytic lymphohistiocytosis
GS: Google Scholar
HGMD: Human Gene Mutation Database
HLH: hemophagocytic lymphohistiocytosis
IFN-γ: interferon-γ
IL: interleukin
NK: natural killer
PolyPhen-2: Polymorphism Phenotyping-2
ROC: receiver operating characteristic
SIFT: Sorting Intolerant From Tolerant
sIL2R: soluble interleukin-2 receptor
SNPs: Single Nucleotide Polymorphisms
TNF-α: tumor necrosis factor-alpha
