# Supplementary figures and images for: Comparison of Th1/Th2 cytokine profiles between primary and secondary haemophagocytic lymphohistiocytosis
Source: Ital J Pediatr. 2016 May 21;42:50. doi: 10.1186/s13052-016-0262-7 (PMC4875745; doi:10.1186/s13052-016-0262-7)

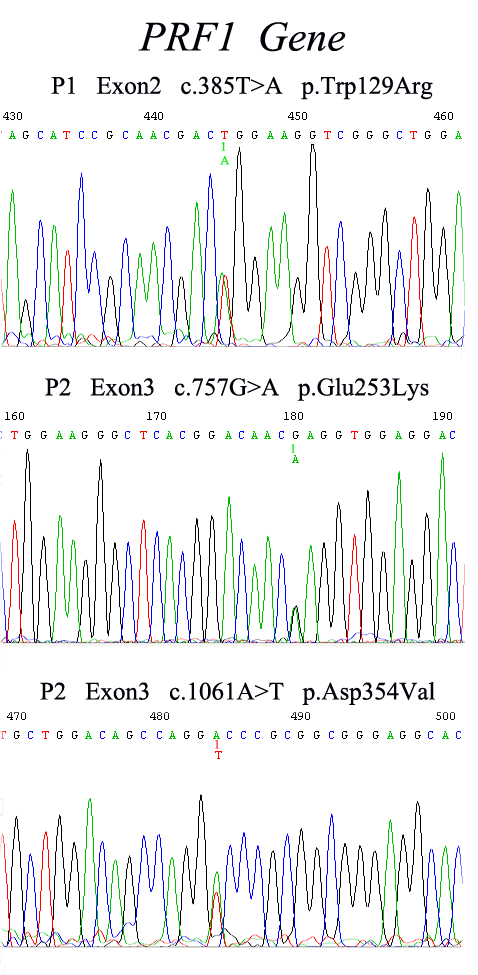

Supplement: Additional file 1: Figure S1. — Sequencing results of PRF1 gene. Genomic DNA sequencing results showed a 1-year-5-month male (P1) had heterozygous mutation c.385T>A (p.Trp129Arg), and a 6-year-3-month female (P2) had compound heterozygous c.757G>A (p.Glu253Lys) and c.1061A>T (p.Asp354Val) of PRF1 gene. (TIF 1391 kb) [file 13052_2016_262_MOESM1_ESM.tif]

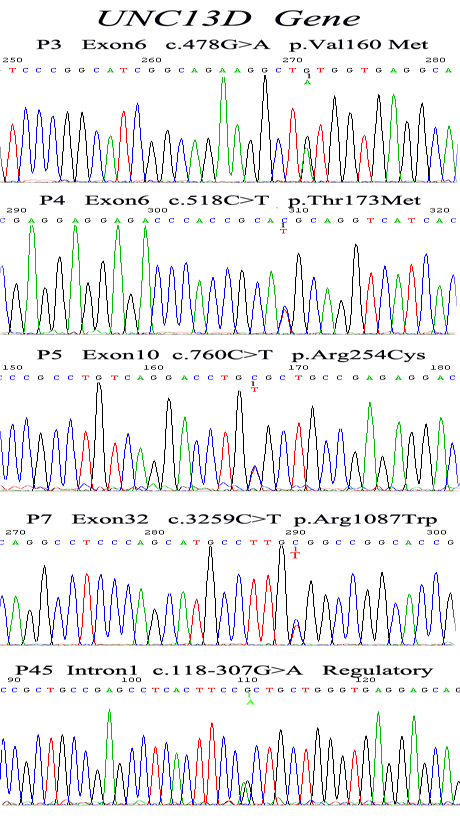

Supplement: Additional file 2: Figure S2. — Sequencing results of UNC13D gene. Five missense mutations of UNC13D gene, c.478G>A (p.Val160 Met) in P3, c.518C>T (p.Thr173Met) in P4, c.760C>T (p.Arg254Cys) in P5, c.3259C>T(p.Arg1087Trp) in P7, and c.118-307G>A in P45, were found in four males and one female, respectively. (TIF 171 kb) [file 13052_2016_262_MOESM2_ESM.tif]

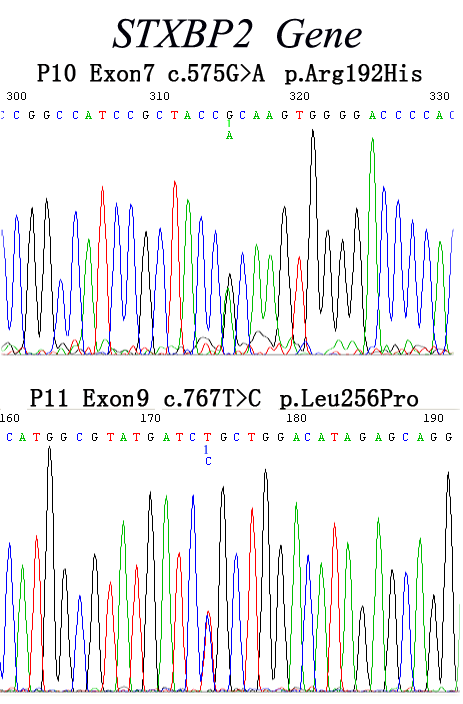

Supplement: Additional file 3: Figure S3. — Sequencing results of STXBP2 gene. Two mutations of STXBP2 gene, c.575G>A (p.Arg192His) in P10, and c.767T>C (p.Leu256Pro) in P11, were found in one male and one female, respectively. (TIF 985 kb) [file 13052_2016_262_MOESM3_ESM.tif]

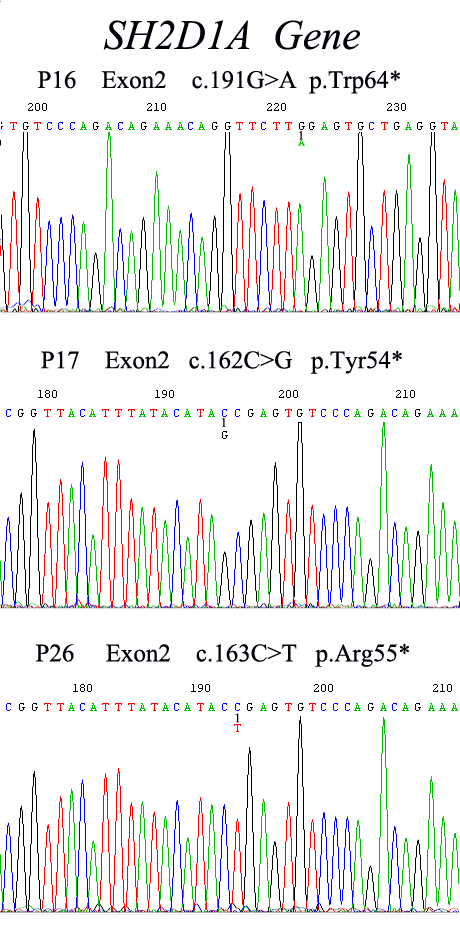

Supplement: Additional file 4: Figure S4. — Sequencing results of SH2D1A gene. Three hemizygous mutations of SH2D1A gene, c.191G> (p.Trp64Ter) in P16, c.162C>G(p.Tyr54Ter) in P17, and c.163C>T(p.Arg55Ter) in P26, were identified in three male patients, respectively. (TIF 136 kb) [file 13052_2016_262_MOESM4_ESM.tif]

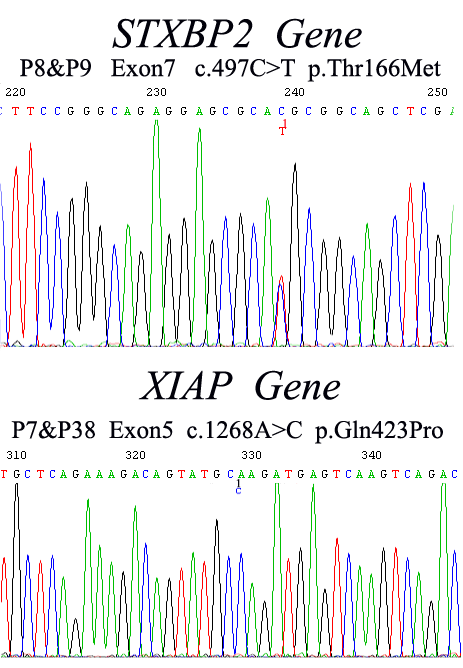

Supplement: Additional file 5: Figure S5. — SNPs of STXBP2 and XIAP genes. Two males patients, 7-year-9-month (P8) and 11-year-11-month (P9), showed c.497C>T (p.Thr166Met) in STXBP2 gene, numbered SNP rs181216956; of XIAP gene, two male patients (P7 and P38) were identified with c.1268A>C(p.Gln423Pro), numbered SNP rs5956583. (TIF 938 kb) [file 13052_2016_262_MOESM5_ESM.tif]

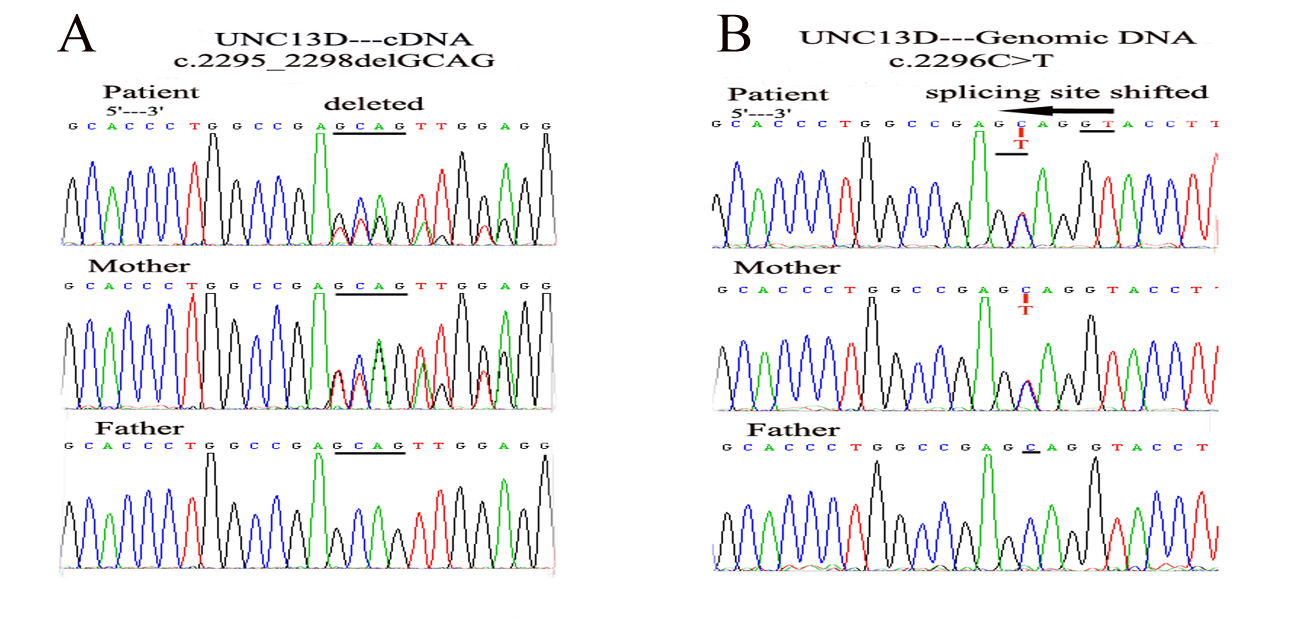

Supplement: Additional file 6: Figure S6. — Sequencing results of UNC13D gene in a Chinese male neonate(P6) and his parents. In mRNA level of P6, the result manifested a heterozygous frameshift mutation c.2295_2298delGCAG (A), which was consistent with the heterozygous point mutation c.2296C>T in genomic DNA level (B). Sequencing results from cDNA and genomic DNA of P6 and his parents both showed that P6 inherited this mutation from his mother. (TIF 391 kb) [file 13052_2016_262_MOESM6_ESM.tif]

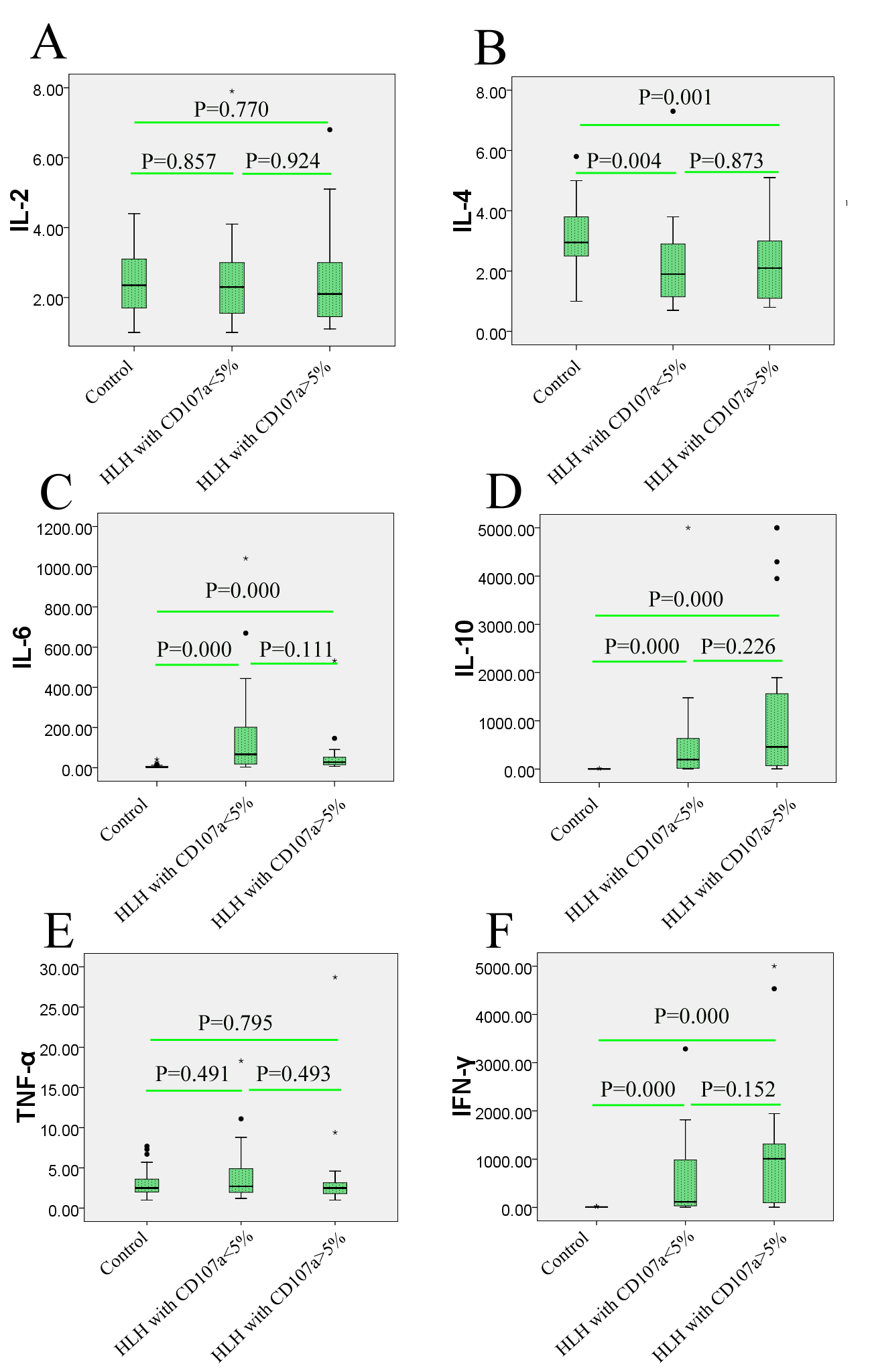

Supplement: Additional file 8: Figure S7. — Comparisons of serum cytokine concentrations (pg/ml) among control, HLH with CD107a<5 %, and HLH with CD107a>5 %. A: IL-2; B: IL-4; C: IL-6; D: IL-10; E: TNF-α; F: IFN-γ. The center horizontal line of the central box is the median (50th percentile), the bottom and top of the box are the 25th and 75th percentiles. The whiskers extend from each end of the box to the 5th and 95th percentiles of the values, respectively. Outliers are the data with values beyond the 5th and 95th percentiles. (TIF 957 kb) [file 13052_2016_262_MOESM8_ESM.tif]
