# Supplementary material for: Comparison of Th1/Th2 cytokine profiles between primary and secondary haemophagocytic lymphohistiocytosis
Source: Ital J Pediatr. 2016 May 21;42:50. doi: 10.1186/s13052-016-0262-7 (PMC4875745; doi:10.1186/s13052-016-0262-7)
Supplement: Additional file 7: Table S1. — Percentage of CD107a expression in NK cells from 43 controls and 36 HLH patients. (DOCX 15 kb) [file 13052_2016_262_MOESM7_ESM.docx]

**Table S1**  Percentage of CD107a expression in NK cells from 43 controls and 36 HLH patients

| **Groups** | **Cases** | **Genetic Mutation Types** | **Percentage of CD107a in NK cells(%)** | **Prognosis** |
| --- | --- | --- | --- | --- |
| Control | n=43 | SNPs or no mutations | Mean(95%CI)=20.9(18.1-23.6) | Alive |
| Primary HLH | P2 | *PRF1* gene compound heterozygous | 32.3 | Alive |
|  | P16 | *SH2D1A* gene hemizygous | 33.8 | Dead |
|  | P17 | *SH2D1A* gene hemizygous | 0.7 | Alive |
|  | P26 | *SH2D1A* gene hemizygous | No done | Alive |
| Secondary HLH | P1 | *PRF1* gene single heterozygous | 3.2 | Alive |
|  | P3 | *UNC13D* gene single heterozygous | 35.1 | Alive |
|  | P4 | *UNC13D* gene single heterozygous | 0.4 | Alive |
|  | P5 | *UNC13D* gene single heterozygous | 5.7 | Dead |
|  | P6 | *UNC13D* gene single heterozygous | 0.5 | Dead |
|  | P7 | *UNC13D* gene single heterozygous  *XIAP* gene SNP | 2.6 | Alive |
|  | P8 | *STXBP2* gene SNP | 3.5 | Alive |
|  | P9 | *STXBP2* gene SNP | 13.4 | Alive |
|  | P10 | *STXBP2* gene single heterozygous | 13.7 | Alive |
|  | P11 | *STXBP2* gene single heterozygous | 4.7 | Alive |
|  | P38 | *XIAP* gene SNP | 0.1 | Alive |
|  | P45 | *UNC13D* gene single heterozygous | 3.1 | Alive |
|  | P12 | No known mutations | No done | Alive |
|  | P13 | No known mutations | 1.6 | Alive |
|  | P14 | No known mutations | No done | Alive |
|  | P15 | No known mutations | No done | Alive |
|  | P18 | No known mutations | No done | Alive |
|  | P19 | No known mutations | 17.5 | Alive |
|  | P20 | No known mutations | No done | Alive |
|  | P21 | No known mutations | 1.2 | Alive |
|  | P22 | No known mutations | 2.2 | Alive |
|  | P23 | No known mutations | 13.9 | Alive |
|  | P24 | No known mutations | No done | Alive |
|  | P25 | No known mutations | No done | Alive |
|  | P27 | No known mutations | 34.2 | Alive |
|  | P28 | No known mutations | 14.9 | Alive |
|  | P29 | No known mutations | 5.3 | Alive |
|  | P30 | No known mutations | 13.2 | Alive |
|  | P31 | No known mutations | No done | Alive |
|  | P32 | No known mutations | 6.1 | Alive |
|  | P33 | No known mutations | 6.9 | Alive |
|  | P34 | No known mutations | 17.4 | Dead |
|  | P35 | No known mutations | 9.9 | Alive |
|  | P36 | No known mutations | 4.5 | Alive |
|  | P37 | No known mutations | 4.2 | Alive |
|  | P39 | No known mutations | 8.1 | Alive |
|  | P40 | No known mutations | 7.5 | Alive |
|  | P41 | No known mutations | 17.0 | Dead |
|  | P42 | No known mutations | 27.0 | Alive |
|  | P43 | No known mutations | 2.3 | Alive |
|  | P44 | No known mutations | 2.6 | Alive |
